# Activation of Natural Killer Cells in Patients with Chronic Bone and Joint Infection due to Staphylococci Expressing or Not the Small Colony Variant Phenotype

**DOI:** 10.1155/2014/280653

**Published:** 2014-03-03

**Authors:** Sébastien Viel, Paul Rouzaire, Frédéric Laurent, Thierry Walzer, Jacques Bienvenu, Florent Valour, Christian Chidiac, Tristan Ferry, The Lyon BJI Study Group

**Affiliations:** ^1^Laboratoire d'Immunologie, Centre Hospitalier Lyon Sud, Hospices Civils de Lyon, 69310 Pierre-Bénite, France; ^2^Université Claude Bernard Lyon 1, 69100 Villeurbanne, France; ^3^Centre International de Recherche en Infectiologie, CIRI, Inserm U1111, CNRS UMR5308, ENS de Lyon, UCBL1, 69007 Lyon, France; ^4^Laboratoire de Bactériologie, Hôpital de la Croix-Rousse, Hospices Civils de Lyon, 69004 Lyon, France; ^5^Centre National de Référence des Staphylocoques, 69008 Lyon, France; ^6^Service de Maladies Infectieuses et Tropicales, Hôpital de la Croix-Rousse, Hospices Civils de Lyon, 93 Grande rue de la Croix-Rousse, 69004 Lyon, France; ^7^Centre Interrégional Rhône-Alpes Auvergne de Référence des IOA Complexes, 69004 Lyon, France

## Abstract

Chronic bone and joint infections (BJI) are devastating diseases. Relapses are frequently observed, as some pathogens, especially staphylococci, can persist intracellularly by expressing a particular phenotype called small colony variant (SCV). As natural killer (NK) cells are lymphocytes specialized in the killing of host cells infected by intracellular pathogens, we studied NK cells of patients with chronic BJI due to staphylococci expressing or not SCVs (10 patients in both groups). Controls were patients infected with other bacteria without detectable expression of SCVs, and healthy volunteers. NK cell phenotype was evaluated from PBMCs by flow cytometry. Degranulation capacity was evaluated after stimulation with K562 cells *in vitro*. We found that NK cells were activated in terms of CD69 expression, loss of CD16 and perforin, in all infected patients in comparison with healthy volunteers, independently of the SCV phenotype. Peripheral NK cells in patients with chronic BJI display signs of recent activation and degranulation *in vivo* in response to CD16-mediated signals, regardless of the type of bacteria involved. This could involve a universal capacity of isolates responsible for chronic BJI to produce undetectable SCVs *in vivo*, which might be a target of future intervention.

## 1. Introduction

Chronic bone and joint infections (BJI) are devastating diseases and staphylococci (*S. aureus* and coagulase-negative staphylococci) are the most frequent bacteria involved in such diseases [1]. Various mechanisms of persistence have been described *in vivo* and *in vitro*, such as biofilm formation (especially in implant-associated infections) and expression of small colony variants (SCVs) [2, 3]. SCVs are a naturally occurring subpopulation of staphylococci, which (i) correspond to a particular fastidious phenotype *in vitro*, expressing slow-growing capacities; (ii) have been described in few bacterial chronic diseases, such as BJI or cystic fibrosis; (iii) are associated with intracellular persistence *ex vivo*; and (iv) are associated with clinical recurrence of the infection. Few data are available about SCVs *in vivo* [2, 4, 5]. In particular, host or bacterial factors that lead to *in vivo* expression of SCVs are unknown.

Natural killer (NK) cells are innate lymphocytes that are specialized in the recognition and killing of host cells infected by intracellular pathogens [6, 7]. Cytotoxicity is mediated *via* the release of prestored granules containing proteins such as perforin and granzymes. NK cell degranulation can be induced through the engagement of various activating receptors, including CD16, the low affinity receptor for the Fc portion of IgG immunoglobulins.

The role of NK cells in BJI has not been investigated. Here, we hypothesized that NK cells may become activated in patients with BJI involving staphylococci expressing the SCV phenotype, as a result of intracellular persistence of the bacteria.

## 2. Material and Methods

We performed a cross-sectional study including 10 immunocompetent patients, with chronic BJI due to staphylococci with SCV phenotype (SCV+ group), defined by typical phenotypic aspect of colonies from peroperative specimen cultures [2]. Patients with chronic BJI were defined as patients with active BJI for over a month. These colonies appear beside the usual colonies in solid culture media, have a slower growing capacity, and appear ~10 times smaller than the parental strain. *S. aureus* SCVs are mostly nonpigmented and are nonhaemolytic, in comparison with the parental strain. To exclude a nonspecific activation of NK cells that may be associated with systemic release of cytokines, only patients without clinical signs of systemic inflammation (defined by (i) body temperature less than 36°C or greater than 38°C; (ii) heart rate >90/min; (iii) respiratory rate >20/min or PaCO2 <32 mmHg; and (iv) white blood cell count <4 × 10^9^/L or >12 × 10^9^/L) were included, and the sampling was done at least 2 weeks after any surgery (cell-mediated immunity could be affected in the course of sepsis and following surgical stress). Control groups included (i) 10 patients with chronic staphylococci BJI without SCV phenotype (SCV− group); (ii) 6 patients with chronic BJI due to other pathogens (other BJI group); and (iii) 19 healthy volunteers (HV). Clinical data such as comorbidity, type of BJI, and the delay between symptoms and bacterial diagnosis were collected. The study was approved by local ethics committee (CAL-2011-21). 5 × 10^5^ peripheral blood mononuclear cells (PBMCs) were isolated using density gradient (MLS Pancoll) and were analyzed for surface CD3, CD8, CD56, CD69, NKG2D, CD16, NKp30, 2B4, and DNAM1 using conjugated monoclonal antibodies (mAbs) (from eBioscience or BD Biosciences) and flow cytometry (FACS Canto II, BD Biosciences). Then, samples were permeabilized using Cytofix/Cytoperm for analyzing intracellular perforin expression. In a separate set of experiments, PBMCs were incubated for 4 hours with or without K562 cells (classical NK cell targets) at a 1 : 1 ratio, as previously described [8]. After one hour of incubation, GolgiStop was added (BD Biosciences). Degranulation (CD107a exposure at the cell surface, measured by using conjugated anti-CD107a mAb) and intracellular IFN*γ* production by NK cells (measured by using conjugated anti-IFN*γ* mAb after cell permeabilization) were analyzed by flow cytometry. Data acquisition was performed using Diva Software and data were subsequently analyzed using Flow Jo software (TreeStar). Statistical analysis was performed using SPSS software version 13 (SPSS Inc., Chicago, IL, USA). Student's *t*-test or nonparametric Mann-Whitney *U* test were used for comparison, as appropriate.

## 3. Results

**Figure 1:**
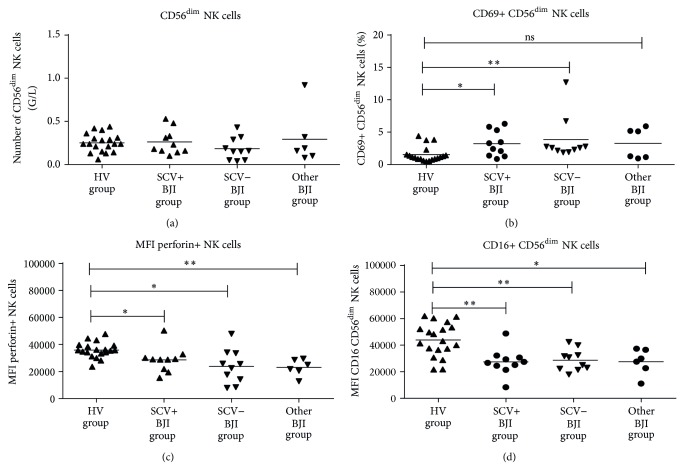
Absolute number of circulating NK cells (a) and their expression of CD69, CD16, and perforin ((b), (c), and (d), resp.) in each group of patients (^*^ *P* < 0.05; ^**^ *P* < 0.001).

**Figure 2:**
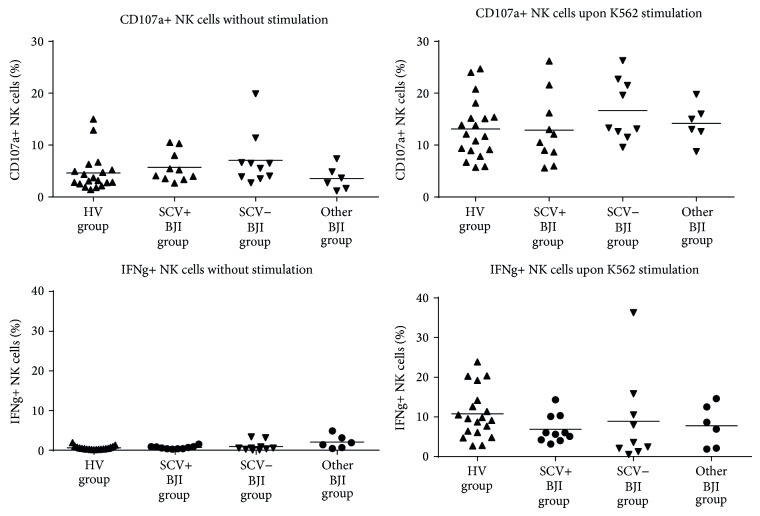
Degranulation and IFN-*γ* production by NK cells before and after stimulation with K562 cells.

After obtaining the patient's consent, peripheral blood sampling was done at a median of 3 months after the diagnosis of chronic BJI. No significant difference between the SCV+ and SCV− groups was observed for the clinical parameters, except for the rate of recurrence, which was significantly higher in the SCV+ group (7/10 versus 0/10, *P* = 0.003) (Table 1). *S. aureus*, in comparison with coagulase-negative staphylococci, was involved in 7/10 and in 5/10 in patients belonging to the SCV+ and SCV− groups, respectively. Patients with other BJI were infected with Enterobacteriaceae (4 patients), *P. acnes* (1 patient), or *P. aeruginosa* (1 patient). Mean number of circulating lymphocytes was similar in all groups (1.99 G/L in HV group; 1.86 G/L in SCV+ BJI group; 1.91 G/L in SCV− BJI group; and 2.35 G/L in other BJI group). We investigated the function and phenotype of circulating CD56^dim⁡^ NK cells, the predominant subset in PBMCs. Their absolute number was similar in all groups (0.22 G/L in HV group; 0.26 G/L in SCV+ BJI group; 0.18 G/L in SCV− BJI group; and 0.29 G/L in other BJI group; Figure 1(a)). We observed an increased expression of CD69 from all staphylococci-infected patients, regardless of the SCV phenotype, indicative of an *in vivo *activation of NK cells (3.2% and 3.8% in SCV+ and SCV− BJI groups, resp.; in comparison with HV group, 2.3%; Figure 1(b)). Similarly, NK cells from all bacteria-infected patients displayed reduced perforin (MFI perforin+ NK cells of 28′589 in SCV+ BJI group; 23′762 in SCV− BJI group; and 23′027 in other BJI group, in comparison with 36′122 in HV group) and CD16 (MFI CD16 CD56^dim⁡^ NK cells of 27′473 in SCV+ BJI group; 28′711 in SCV− BJI group; and 27′568 in other BJI group, in comparison with 40′414 in HV group) expression that could be due to CD16-dependent *in vivo* cytotoxicity (Figures 1(c) and 1(d), resp.). The level of different other NK cell receptors (NKG2D, NKp30, DNAM1, and 2B4) was similar in all groups. Moreover, in response to stimulation with K562 cells, degranulation (12.9%, 16.7%, and 14.2% of CD107a+ NK cells in SCV+, SCV−, and other BJI groups, resp.) and IFN-*γ* production (6.9%, 8.9%, and 9.7% of IFN*γ*+ NK cells in SCV+, SCV−, and other BJI groups, resp.) by NK cells were found to be similar in all patient groups (Figure 2).

## 4. Discussion

The host immune response during chronic BJI is poorly documented, especially when bacteria with a SCV phenotype are involved. Indeed, the detection of SCV is infrequent in clinical practice, and as SCVs are associated with relapse and complex cases, performing a study on the host response during chronic BJI due to staphylococci expressing the SCV phenotype is only restricted to tertiary university hospitals considered as reference center for the care of BJI and requires a multidisciplinary approach. This study was designed to better understand the immune response occurring in patients with BJI and to decipher the host parameters leading to the SCV phenotype of the infecting bacteria. NK cells are involved in the innate immune response against intracellular pathogens [7]. Altogether, our results demonstrate that circulating NK cells show signs of recent activation and cytolytic activity in patients with chronic BJI, regardless of the SCV phenotype of the bacteria involved. Few data are available on the role of NK cells in the control of bacterial infections, especially of staphylococcal infections. Some bacteria, such as *S. enteritica*, *M. tuberculosis*, and Staphylococci (by expressing SCVs), have the ability to invade and persist intracellularly in host cells [2, 6, 9]. Lapaque et al. showed that NK cell activation *in vitro* led to a dramatic reduction in the numbers of intramacrophagic *S. enteritica* and was involved in the clearance of the pathogen [6]. More recently, Kee et al. showed that circulating NK levels and function were reduced in patients with *M. tuberculosis* infection in comparison with patients with latent infection and in comparison with healthy volunteers [9]. Focusing on NK cells and staphylococcal infections, Small et al. demonstrated in a mouse model that NK cells play a critical protective role against *S. aureus* lung infection [10]. The response of NK cells in chronic BJI is however unknown. Here, we show that peripheral NK cells in patients with chronic BJI are activated and show signs of recent degranulation *in vivo*, regardless of the type of bacteria involved and of their SCV phenotype. This suggests that NK cells play a role in the defense against a broad range of bacteria, whether they display intracellular cycles or not. However, staphylococci expressing the SCV phenotype have the ability to revert rapidly to the normal phenotype in culture media [2, 5]. Thus, some staphylococci not expressing the SCV phenotype *in vitro* might in fact express this phenotype *in vivo*. Moreover, SCVs are known to be more fastidious in culture, than the parental strain, and could be underestimated. As a consequence, some staphylococci, not expressing the SCV phenotype *in vitro*, might in fact express this phenotype *in vivo*. Furthermore, expression of SCVs is not only described with Staphylococci, but also with *P. aeruginosa*, especially in patients with cystic fibrosis, or with other bacteria such as *Enterococcus* spp.[11, 12]. As our results indicate that NK cells were activated in patients with chronic BJI regardless of the type of bacteria involved, production of SCVs (i.e., invasion and intracellular persistence at the site of infection) could therefore be a common process in the pathophysiology of chronic BJI.

Our study also shows that NK cells in patients with BJI have a decreased expression of CD16 and a normal expression of all other NK cell receptors tested. As CD16 is involved in ADCC, this suggests that NK cells may kill bacteria-infected cells covered with antibodies *in vivo*. Such cells could be infected osteoblasts or other immune cells such as monocytes/macrophages. Further studies are required to elucidate this point.

Here, we report that peripheral NK cells in patients with chronic BJI display signs of recent activation and degranulation *in vivo* in response to CD16-mediated signals, regardless of the type of bacteria involved and of their SCV phenotype. This could involve a universal capacity of isolates responsible for chronic BJI to produce SCVs, which might be a target of future intervention. For instance, new drugs (acetyldepsipeptides) that have the ability to kill “dormant bacterial cells” have been discovered, and their use in combination with classical antimicrobial therapy might limit recurrence and relapse in patients with chronic BJI [13].

## Figures and Tables

**Table 1:** 

	Patients with SCV+ (*n* = 10)	Patients with SCV− (*n* = 10)	Patients with other BJI (*n* = 6)	Total (*n* = 26)	*P∗*
Age (median, years)	61 (52–79)	57 (47–69)	57 (33–69)	62 (47–71)	0.247
Male sex (*n*, %)	6 (60)	5 (50)	4 (67)	15 (58)	0.653
Diabetes mellitus (*n*, %)	4 (40)	1 (10)	2 (33)	7 (27)	0.303
Charlson's Comorbidity Index >2 (*n*, %)	6 (60)	5 (50)	3 (50)	17 (65)	0.656
Implant-associated infection (*n*, %)	9 (90)	10 (100)	4 (67)	23 (89)	1
Recurrence (*n*, %)	7 (70)	0 (0)	3 (50)	10 (39)	0.003
Delay between symptoms and bacterial diagnosis (median, days)	133 (61–209)	129 (13–88)	129 (32–904)	73 (24–177)	0.082
Plurimicrobial infection (*n*, %)	1 (10)	2 (20)	1 (17)	4 (15)	1
Bacterial growth at 48 h (*n*, %)	6 (60)	6 (60)	4 (80)	16 (64)	1
Delay between bacterial diagnosis and blood sampling (median, days)	93 (20–248)	52 (15–121)	202 (67–379)	87 (26–257)	0.356
Delay between last surgery and blood sampling (median, days)	47 (20–178)	52 (12–121)	202 (47–10523)	58 (20–214)	0.780

*Note*. SCV: small colony variant; BJI: bone and joint infection; ∗resulting from the comparison between SCV+ group and SCV− group.
